# Cardiovascular Risk in Xavante Indigenous Population

**DOI:** 10.5935/abc.20180090

**Published:** 2018-07

**Authors:** Luana Padua Soares, Amaury Lelis Dal Fabbro, Anderson Soares Silva, Daniela Saes Sartorelli, Luciana Ferreira Franco, Patrícia Chamadoira Kuhn, Regina Santiago Moises, João Paulo Botelho Vieira-Filho, Laércio Joel Franco

**Affiliations:** 1 Faculdade de Medicina de Ribeirão Preto - Universidade de São Paulo, São Paulo, SP - Brazil; 2 Universidade Federal de Uberlândia, Uberlândia, MG - Brazil; 3 Escola Paulista de Medicina - Universidade Federal de São Paulo, São Paulo, SP - Brazil

**Keywords:** Cardiovascular Diseases / epidemiology, Risk Factors, Indigenous Population, Adult, Obesity, Dyslipidemias

## Abstract

**Background:**

The prevalence of cardiovascular risk factors is little known in Brazilian indigenous populations. In the last two decades, important changes have occurred in the lifestyle and epidemiological profile of the Xavante people.

**Objective:**

to assess the prevalence of cardiovascular risk factors in Xavante adults in São Marcos and Sangradouro/Volta Grande reserves, in the state of Mato Grosso, Brazil.

**Methods:**

Cross-sectional study carried out with 925 Xavante people aged ≥ 20 years between 2008 and 2012. The following indicators were assessed: triglycerides (TG), total, LDL and HDL-cholesterol, Castelli index I and II, TG/HDL-cholesterol ratio, apo B / Apo A1 ratio, Framingham risk score, C-reactive protein, body mass index (BMI), waist circumference (WC), hypertriglyceridemic waist (HW), glycemia and blood pressure. Kolmogorov-Smirnov, Student's t test and Chi-square test (χ^2^) were used for statistical analysis, and significance level was set at 5%.

**Results:**

High prevalence of elevated cardiovascular risk was observed in men and women according to HDL-cholesterol (66.2% and 86.2%, respectively), TG (53.2% and 51.5%), TG/HDL-cholesterol ratio (60.0% and 49.1%), C-reactive protein (44.1% and 48.1%), BMI (81.3% and 81.7%), WC (59.1% and 96.2%), HW (38.0% and 50,6%) and glycemia (46.8% and 70.2%). Individuals aged 40 to 59 years had the highest cardiovascular risk.

**Conclusions:**

The Xavante have a high cardiovascular risk according to several indicators evaluated. The present analysis of cardiovascular risk factors provides support for the development of preventive measures and early treatment, in attempt to minimize the impact of cardiovascular diseases on this population.

## Introduction

Cardiovascular diseases (CVDs) are the main cause of mortality and morbidity in Brazil and the world. Approximately one third of deaths are caused by CVDs. Besides, they constitute one of the main causes of long hospital stay and health costs in Brazil.^1,2^

Most CVDs result from unhealthy lifestyle and modifiable risk factors. Altered lipid profile, diabetes mellitus, smoking, advanced age, family history, sedentary lifestyle and weight excess are the main predisposing factors for CVDs.^1-3^ CVDs start in early stages of life and progress silently until first manifestations in advanced stages. The earlier the risk factors are identified, the higher the possibility of prevention to prevent and reduce complications.^2^

The prevalence of cardiovascular risk factors is still poorly investigated in indigenous populations in Brazil. In the last decades, considerable changes in eating habits and physical activity level have occurred in Xavante people, contributing to increased prevalence of non-communicable chronic diseases in this population.^4,5^ However, despite significant literature on health conditions, there are no studies on cardiovascular risk in this indigenous group.

Considering that CVDs increase the risk of premature deaths, disabilities and decreased quality of life, and exert an economic impact for families, communities and society, determining the prevalence of cardiovascular risk factors would be valuable for the establishment of prevention strategies.^2^

The aim of this study was to evaluate the prevalence of cardiovascular risk factors in Xavante adults from São Marcos and Sangradouro/Volta Grande indigenous reserves in Mato Grosso state, Brazil.

## Methods

This was a cross-sectional study of Xavante adults living in São Marcos and Sangradouro/Volta Grande indigenous reserves in Mato Grosso State, Brazil.

The study was approved by the Research Ethics Committee of Hospital das Clínicas da Faculdade de Medicina de Ribeirão Preto - USP, Escola Paulista de Medicina - UNIFESP, CONEP and FUNAI.

Xavante communities live in eight indigenous reserves located in Mato Grosso State, Brazil. The study was conducted by periodic visits made to these communities from October 2008 to January 2012. Total population of indigenous in these reserves is estimated to be 4,020 people, 1,582 of them aged 20 years or older.^6^ All subjects aged 20 years or older were invited to participate in the study.

Physical examination, including anthropometry, and collection of blood samples were performed in the villages. After being informed about the study objectives, the tribe chiefs and participants gave their consent, mostly written. To illiterate participants (14%), the consent forms were read by community health agents, and fingerprints were used to confirm their agreement to participate in the study.

The following variables were assessed: sex, age, weight (Kg), height (m), waist circumference (WC) (cm), triglyceride levels (TG) (mg/dL), total cholesterol (TC) (mg/dL), low-density lipoprotein cholesterol (LDL-c), high-density lipoprotein cholesterol (HDL-c), apolipoproteins A1 and B (apo 1 and apo B) (mg/dL), capillary blood glucose levels at baseline and at 2 hours (mg/dL), systolic and diastolic arterial pressure (mm/Hg), high-sensitivity C-reactive protein (hs-CRP) (mg/dL).

Body weight was measured using an electronic scale (Plenna®), with maximum capacity of 150 Kg, and height was measured using a portable stadiometer (Alturexata®). Weight and height values were used for body mass index (BMI) calculation (weight (kg)/height(m)^2^].^7^ WC was measured using an inelastic measuring tape at the midpoint between the lowest rib and iliac crest, at standing position.

Venous blood was collected after an 8-10 hour fast, using sterile disposable tubes (Vacutainer ®). Samples were stored at -20ºC and transported to a laboratory in Sao Paulo, Brazil. Measurements of serum TG, TC, LDL-c, HDL-c, apo A1 and apo B were determined by enzymatic methods, and hs-CRP levels were determined by immunoturbidimetry.

Blood pressure (BP) was measured on the left arm in the sitting position after 5 minutes at rest, using an automatic digital monitor (OMRON HEM-742INTC®). Measurements were taken three times, and the mean of the last two measurements was considered for analysis.

Capillary blood glucose at baseline and two hours after a 75 g anhydrous glucose overload (Glutol®) were measured using a portable glucose meter (HemoCue® Glucose 201, HemoCue AB).

Castelli index I (TC/HD/l-c ratio) and II (LDL-c/HDL-c ratio)^8^, TG/HDL-c,^9^ ApoB/ApoA1^10^ and Framingham risk score^11^ were calculated. Hypertriglyceridemic waist (HW) was defined as the simultaneous presence of increased WC and increased TG levels.^12^

Indicators of cardiovascular risk used in the study are described in Chart 1.^7-17^

**Chart 1:** Cardiovascular risk indicators

Indicators	RISK
Total cholesterol (mg/dl)^13^	≥ 200 mg/dl
HDL-cholesterol (mg/dl)^13^	< 50 mg/dl in women and < 40 mg/dl in men
LDL-cholesterol (mg/dl)^13^	≥ 130 mg/dl
Triglycerides (mg/dl)^13^	≥ 150 mg/dl
Castelli index I^8^	> 4.4 for women and > 5.1 for men
Castelli index II^8^	> 2.9 for women and > 3,3 for men
TG/HDL-C ratio^9^	≥ 3.8
ApoB/ApoA1 ratio^10^	> 0.8 for women and > 0.9 for men
Framingham risk score^11^	Low risk – probability < 10%
	Intermediate risk – probability between 10% and 20%
	High risk – probability > 20%
Hs-CRP (mg/L)^14^	Low risk - < 1.0 mg/L
	Intermediate risk – 1.0 – 3.0 mg/L
	High risk - >3.0 mg/L
BMI (kg/m^2^)^7,15^	≥ 25.0 kg/m^2^ for adults
	≥ 27.0 kg/m^2^ for elderly subjects
Waist circumference (cm)^7^	≥ 94 cm in men and ≥ 80 cm in women
Hypertriglyceridemic waist^7,13^	Increased WC (≥ 94 cm in men and ≥ 80 cm in women) and TG ≥ 150 mg/dl
Glycemia (mg/dL)^16^	Casual glucose level ≥ 200 mg/dL and/or
	Glucose after 2 hours ≥ 140 mg/dL and/or
	using oral antidiabetic drugs or insulin
Blood pressure (mm/Hg)^17^	Systolic arterial pressure ≥ 140mmHg and/or
	Diastolic arterial pressure ≥ 90mmHg and/or
	Use of anti-hypertensive agents

WC: waist circumference; TG: triglycerides; hs-CRP: high sensitivity C-reactive protein; BMI: body mass index

### Statistical analysis

The *Kolmogorov-Smirnov* test was used to test normality of variable distributions. Continuous variables were described as mean and standard deviation, and Student's t-test was used to compare the variable means between men and women. Categorical variables were expressed as absolute and relative frequencies, and the chi-square test (χ^2^) was used for comparison of proportions. Analyses were formed using the *Statistical Package for Social Sciences* (SPSS) software version 17, and significance level was set at 5%.

## Results

Study population was composed of 925 Xavante people, 455 (49.2%) men and 470 (50.8%) women. Most (57.0%) of them were aged between 20 and 39 years.

Cardiovascular risk indicators are presented as mean and standard deviations in Table 1. Mean apo A1, WC, BMI and glucose levels were higher in women than men, whereas mean Castelli index I and II, Framingham score, Apo B/Apo A-I ratio and systolic and diastolic BP were higher in men than in women.

**Table 1:** Cardiovascular risk indicators (mean and standard deviation) by sex in Xavante adults in Sao Marcos and Sangradouro reserves, Brazil, 2008-2012

Variables	Mean ± SD			p-value*
	Total	Women	Men	
Age (years)	42.8 ± 19.2	42.5 ± 19.4	43.2 ± 19.0	0.586
Total cholesterol (mg/dl)	146.4 ± 43.1	146.8 ± 43.2	146.0 ± 43.0	0.757
HDL-cholesterol (mg/dl)	38.9 ± 8.0	40.6 ± 8.2	37.1 ± 7.5	< 0.001
LDL-cholesterol (mg/dl)	70.4 ± 24.6	70.0 ± 23.3	70.8 ± 26.0	0.621
Triglycerides (mg/dl)	199.1 ± 171.2	196.4 ± 180.0	202.1 ± 161.7	0.615
Castelli index I (CT/HDL-c)	3.9 ± 1.3	3.7 ± 1.3	4.0 ± 1.3	< 0.001
Castelli index II (LDL-c/HDL-c)	1.8 ± 0.7	1.8 ± 0.6	2.0 ± 0.8	< 0.001
TG/HDL-C ratio	5.4 ± 5.1	5.2 ± 5.3	5.7 ± 4.8	0.107
Framingham risk score	5.7 ± 6.5	5.1 ± 6.8	6.3 ± 6.1	0.006
Apo B (mg/dl)	72.9 ± 18.9	73.2 ± 17.8	72.5 ± 17.9	0.577
Apo A1 (mg/dl)	106.8 ± 4.7	110.1 ± 14.4	103.4 ± 14.1	< 0.001
ApoB/ApoA1 ratio	0.69 ± 0.18	0.67 ± 0.16	0.71 ± 0.18	0.001
High-sensitivity C-reactive protein	6.1 ± 11.6	6.3 ± 12.7	5.8 ± 10.3	0.543
Waist circumference (cm)	97.3 ± 10.9	98.6 ± 11.1	95.9 ± 10.4	< 0.001
Body mass index (kg/m^2^)	30.3 ± 5.1	30.7 ± 5.6	29.9 ± 4.6	0.011
Baseline glucose level (mg/dL)	152.5 ± 104.9	163.7 ± 112.4	140.8 ± 95.3	0.001
Glucose level at 2 hours (mg/dL)	148.9 ± 51.8	158.6 ± 49.0	140.2 ± 52.8	< 0.001
Diastolic blood pressure (mm/Hg)	72.7 ± 10.8	71.5 ± 10.6	74.0 ± 10.9	< 0.001
Systolic blood pressure (mm/Hg)	122.3 ± 17.4	119.7 ± 18.4	125.1 ± 15.8	< 0.001

* Student’s t-test; LDL: low-density lipoprotein; HDL: high-density lipoprotein.

We found a high prevalence of elevated cardiovascular risk according to HDL-c, TG, TG/HDL-c ratio, CRP-hs, BMI, WC, HW and glucose levels, although a small number of participants had increased levels of TC or LDL-c. In general, participants aged between 40 and 59 years were the most exposed to cardiovascular risk factors (Tables 2 and 3).

**Table 2:** Frequency of cardiovascular risk factors by age range in Xavante women in São Marcos and Sangradouro reserves, Brazil, 2008-2012

Cardiovascular risk indicators	20 – 39 years	40 – 59 years	≥ 60 years	Total	p-value*
**Total cholesterol (mg/dl)**					**0.039**
Normal	254 (95.5)	94 (94.9)	93 (88.6)	441 (93.8)	
Risk	12 (4.5)	5 (5.1)	12 (11.4)	29 (6.2)	
**HDL-cholesterol (mg/dl)**					**0.015**
Normal	38 (14.3)	6 (6.1)	21 (20.0)	65 (13.8)	
Risk	228 (85.7)	93 (93.9)	84 (80.0)	405 (86.2)	
**LDL-cholesterol (mg/dl)**					**0.620**
Normal	254 (99.2)	86 (98.9)	98 (98.0)	438 (98.9)	
Risk	2 (0.8)	1 (1.1)	2 (2.0)	5 (1.1)	
**Triglycerides (mg/dl)**					**< 0.001**
Normal	161 (60.5)	31 (31.3)	36 (34.3)	228 (48.5)	
Risk	105 (38.5)	68 (68.7)	69 (65.7)	242 (51.5)	
**Castelli index I**					**0.054**
Normal	230 (86.5)	81 (81.8)	80 (76.2)	391 (83.2)	
Risk	36 (13.5)	18 (18.2)	25 (23.8)	79 (16.8)	
**Castelli index II**					**0.571**
Normal	247 (96.5)	82 (94.3)	97 (97.0)	426 (96.2)	
Risk	9 (3.5)	5 (5.7)	3 (3.0)	17 (3.8)	
**TG/HDL-C ratio**					**< 0.001**
Normal	160 (60.2)	35 (35.4)	44 (41.9)	239 (50.9)	
Risk	106 (39.8)	64 (64.6)	61 (58.1)	231 (49.1)	
**ApoB/ApoA1 ratio**					**0.018**
Normal	242 (91.3)	85 (85.7)	85 (81.0)	411 (87.8)	
Risk	23 (8.7)	14 (14.3)	20 (19.0)	57 (12.2)	
**Framingham**					**< 0.001**
Low risk	266 (100.0)	85 (85.9)	29 (27.6)	380 (80.9)	
Intermediate risk	0 (0.0)	12 (12.1)	51 (48.6)	63 (13.4)	
High risk	0 (0.0)	2 (2.0)	25 (23.8)	27 (5.7)	
**CRP (mg/L)**					**0.650**
Low risk	40 (15.0)	11 (11.1)	17 (16.2)	68 (14.5)	
Intermediate risk	102 (38.3)	34 (34.3)	40 (38.1)	176 (37.4)	
High risk	124 (46.6)	54 (54.5)	48 (45.7)	226 (48.1)	
**BMI (kg/m^2^)**					**< 0.001**
Normal	25 (9.4)	7 (7.1)	54 (51.4)	86 (18.3)	
Risk	241 (90.6)	92 (92.9)	51 (48.6)	384 (81.7)	
**Waist circumference (cm)**					**0.071**
Normal	12 (4.5)	0 (0.0)	6 (5.7)	18 (3.8)	
Risk	254 (95.5)	99 (100.0)	99 (94.3)	452 (96.2)	
**Hypertriglyceridemic waist**					**<0.001**
Normal	162 (60.9)	31 (31.3)	39 (37.1)	232 (49.4)	
Risk	104 (39.1)	68 (68.7)	66 (62.9)	238 (50.6)	
**Glycemia (mg/dL)**					**< 0.001**
Low risk	104 (39.1)	15 (15.2)	21 (20.0)	140 (29.8)	
High risk	162 (61.9)	84 (84.8)	84 (80.0)	330 (70.2)	
**Blood pressure (mm/Hg)**					**< 0.001**
Low risk	254 (95.5)	76 (76.8)	71 (67.6)	401 (85.3)	
High risk	12 (4.5)	23 (23.2)	34 (32.4)	69 (14.7)	

* Chi square test (χ^2^); TG: triglycerides; BMI: body mass index LDL: low-density lipoprotein; HDL: high-density lipoprotein.

**Table 3:** Frequency of cardiovascular risk factors by age range in Xavante men in São Marcos and Sangradouro reserves, Brazil, 2008-2012

Cardiovascular risk indicators	20 – 39 years	40 – 59 years	≥ 60 years	Total	p-value*
**Total cholesterol (mg/dl)**					**0.871**
Normal	238 (91.2)	100 (90.9)	78 (92.9)	416 (91.4)	
Risk	23 (8.8)	10 (9.1)	6 (7.1)	39 (8.6)	
**HDL-cholesterol (mg/dl)**					**0.035**
Normal	78 (29.9)	38 (34.5)	38 (45.2)	154 (33.8)	
Risk	183 (70.1)	72 (65.5)	46 (54.8)	301 (66.2)	
**LDL-cholesterol (mg/dl)**					**0.448**
Normal	242 (98.8)	93 (96.9)	77 (98.7)	412 (98.3)	
Risk	3 (1.2)	3 (3.1)	1 (1.3)	7 (1.7)	
**Triglycerides (mg/dl)**					**0.003**
Normal	120 (46.0)	41 (37.3)	52 (61.9)	213 (46.8)	
Risk	141 (54.0)	69 (62.7)	32 (38.1)	242 (53.2)	
**Castelli index I**					**0.128**
Normal	225 (86.2)	94 (85.5)	79 (94.)	398 (87.5)	
Risk	36 (13.8)	16 (14.5)	5 (6.0)	57 (12.5)	
**Castelli index II**					**0.033**
Normal	227 (92.7)	94 (97.9)	77 (98.7)	398 (95.0)	
Risk	18 (7.3)	2 (2.1)	1 (1.3)	21 (5.0)	
**TG/HDL-C ratio**					**< 0.001**
Normal	98 (37.5)	35 (31.8)	49 (58.3)	182 (40.0)	
Risk	163 (62.5)	75 (68.2)	35 (41.7)	274 (60.0)	
**ApoB/ApoA1 ratio**					**0.128**
Normal	229 (88.4)	102 (92.7)	79 (95.2)	410 (90.7)	
Risk	30 (11.6)	8 (7.3)	4 (4.8)	42 (9.3)	
**Framingham**					**< 0.001**
Low risk	261 (100.0)	79 (71.8)	1 (1.2)	34 (74.9)	
Intermediate risk	0 (0.0)	24 (21.8)	21 (25.0)	45 (9.9)	
High risk	0 (0.0)	7 (6.4)	62 (73.8)	69 (15.2)	
**Hs-CRP (mg/L)**					**0.867**
Low risk	47 (18.0)	19 (17.3)	17 (20.5)	83 (18.3)	
Intermediate risk	102 (39.1)	42 (38.2)	27 (32.5)	171 (37.7)	
High risk	112 (42.9)	49 (44.5)	39 (47.0)	200 (44.1)	
**BMI (kg/m^2^)**					**< 0.001**
Normal	33 (12.6)	10 (9.1)	42 (50.0)	85 (18.7)	
Risk	228 (87.4)	100 (90.9)	42 (50.0)	370 (81.3)	
**Waist circumference (cm)**					**< 0.001**
Normal	118 (45.2)	27 (24.5)	41 (48.8)	186 (40.9)	
Risk	143 (54.8)	83 (75.5)	43 (51.2)	269 (59.1)	
**Hypertriglyceridemic waist**					**0.001**
Normal	164 (62.8)	54 (49.1)	64 (76.2)	282 (62.0)	
Risk	97 (37.2)	56 (50.9)	20 (23.8)	173 (38.0)	
**Glycemia (mg/dL)**					**< 0.001**
Low risk	160 (61.3)	46 (41.8)	36 (42.9)	242 (53.2)	
High risk	101 (38.7)	64 (58.2)	48 (57.1)	213 (46.8)	
**Blood pressure (mm/Hg)**					**< 0.001**
Low risk	236 (90.4)	86 (78.2)	51 (60.7)	373 (82.0)	
High risk	25 (9.6)	24 (21.8)	33 (39.3)	82 (18.0)	

* Chi square test (χ^2^); TG: triglycerides; hs-CRP: high sensitivity C-reactive protein; BMI: body mass index; LDL: low-density lipoprotein; HDL: high-density lipoprotein.

## Discussion

Our findings show that Xavante people have an increased risk for CVDs according to HDL-c, TG, TG/HDL-c ratio, CRP-hs, BMI, WC, HW and glucose levels. Based on this, the prevalence of these diseases and consequently the risk of death, disabilities, and reduced quality of life may increase in this population in the next years.

Although several methods and indicators may be used to estimate cardiovascular risk, none of them can predict cardiovascular risk alone, and hence, should be evaluated together.

One of the cardiovascular risk factors evaluated in our study was lipid profile. The risk for atherosclerotic disease is associated with increased TC and LDL-c levels and low HDL-c levels.^13^ With respect to TG, however, there is no consensus on whether they are a direct cause of atherosclerosis or a marker of other high-risk conditions.^18^ Only a small percentage of Xavante people had increased TC and LDL-c levels. Nevertheless, similarly to other indigenous populations,^19,20^ the Xavantes showed a high prevalence of increased TG and decreased HDL-c levels.

Castelli index I (CT/HDL-c) and II (LDL-c/HDL-c) and the TG/HDL-c ratio have been used to assess the combined influence of cardiovascular risk factors.^8,9^ We did not find an increased cardiovascular risk according to these indexes in the study population; however, values of TG/HDL-c ratio in 49.1% of women and 60.0% of men were indicative of high cardiovascular risk, corroborating the increased levels of TG and decreased levels of HDL-c observed in the population.

Plasma apolipoproteins A1 and B and the apo B/apo A1 ratio have been described as the best predictors of cardiovascular risk as compared with lipid and lipoprotein levels or the Castelli index I and II.^21,22^ Apolipoproteins are structural and functional components of lipoproteins. Apo A1 constitutes non-atherogenic lipid fractions (HDL-c), whereas apo B constitutes atherogenic ones (chylomicrons, LDL, IDL and VLDL). Thus, apo B/apo A1 ratio represents the balance between atherogenic and antiatherogenic lipoproteins.^21,22^ Increased apo B and apoB/A1 and reduced apo A1 levels have been consistently associated with risk for CVDs.^22^ In our study group, 12.2% of women and 9.3% of men had an apo B/apo A1 ratio indicative of cardiovascular risk. We have not found any studies evaluating these indicators in other indigenous populations.

CRP, an acute-phase protein released into blood in response to inflammatory cytokines and a biomarker of systemic inflammation, was also evaluated in the current study. Increased CRP levels have been associated with coronary disease and stroke, even in patients with normal lipid profile.^14^ Approximately half of Xavante people had CRP-hs levels indicative of high cardiovascular risk. However, caution is needed in interpreting these data, as other inflammatory diseases can also increase CRP levels. Infectious and parasite diseases are common in indigenous populations, including the Xavante people, which may have influenced the results.

Framingham score is one of the algorithms used in detecting the risk for CVDs.^11^ In our study, 15.2% of men and 5.7% of women have increased risk of developing CVDs in the next 10 years according to this score. Although this score has been developed for subjects aged 30 years or older, in the current study, patients aged between 20 and 29 years were also included, corresponding to 28.0% of the study population. In the "age" component of Framingham score calculation, these subjects received the rating assigned for individuals aged between 30 and 34 years (zero). No participant aged between 20 and 39 years showed increased cardiovascular risk. Despite its high predictive value, Framingham score does not consider weight excess or sedentary lifestyle, both considered important cardiovascular risks.^23^

Studies have reported a considerable increase in the prevalence of overweight and obesity in indigenous populations.^4,5,24^ Studies conducted in specific populations have shown a high proportion of overweight and obese adults, greater than 50% in some age groups.^25-27^

Obesity is an important risk factor for CVDs. It is independently associated with risk for coronary disease, atrial fibrillation and heart failure. On the other hand, obesity, particularly abdominal or visceral obesity, is associated with other factors known to increase cardiovascular risk, such as systemic arterial hypertension (SAH), diabetes mellitus, hypertriglyceridemia and low HDL-c.^23^

More recently, HW has also been used as an indicator of cardiometabolic risk. HW is defined as the simultaneous presence of increased WC and increased TG levels, and may be used in the screening of patients likely to have the atherogenic metabolic triad - fasting hyperinsulinemia; hyperapolipoprotein B; and high proportion of small, dense LDL-c. For this reason, HW has been used as a practical, viable, low-cost tool in the identification of patients with high cardiovascular risk.^12,28^ The prevalence of HW found in our study group (50.6% in women and 38.0% in men) was higher than that reported in other Brazilian studies.^29,30^

Diabetic subjects have from twice to three times the risk to suffer a cardiovascular event.^31^ Besides, cardiovascular and cerebrovascular diseases are important causes of death in diabetes mellitus patients, accounting for up to 80% of deaths.^32,33^

Altered glucose levels is a health problem of large magnitude in Xavante people. In the present study, 70.2% of women and 46.8% of men had diabetes and decreased glucose tolerance, indicating that they constitute a vulnerable group. This was a much higher prevalence as compared with that in the Brazilian population.^34^

SAH is also an important risk factor for CVDs.^17^ The prevalence of SAH in Xavante people - 14.7% in women and 18.0% in men - was lower than mean values reported in Brazilian adult populations, ranging from 20.0%^35^ to 24.1%.^36^

As compared with the Xavantes of Pimentel Barbosa reserve, there was a tendency of increase in the prevalence of SAH. In 1962, no cases of HAS was observed in this population.^37^ In 2009, however, the prevalence reached 8.1% among men and 5.8% among women.^38^ This may result from social, cultural, economic and environmental changes in Xavante people, that culminated in reduction of physical activity and changes in eating habits with increased consumption of packaged foods high in sugar, fat and sodium.^4,27^

This study has some limitations. Despite the large sample size, it corresponded to only 60% of the total estimated subjects aged 20 years or older in these communities, suggesting a selection bias, since healthier individuals tend to be less interested in participating in the study. In addition, some smaller, less accessible indigenous communities were not included in the study, affecting the participation rate. Limitations regarding communication between indigenous people and investigators, which may have been a source of bias, were partly prevented by participation of health professionals, members of the indigenous community in data collection. Also, due to cultural differences, we cannot assure that all volunteers were in fasting conditions on blood collection day despite instructions to do so; in addition to a more irregular eating pattern, they may have not understood the importance of such condition for laboratory tests. Thus, caution is need in interpreting TG levels and TG/HDL ratio and HW values. Another limitation was the fact that we did not evaluate smoking habit, which is a key cardiovascular risk factor, not only isolated but also as a Framingham score component. All subjects were rated as non-smokers in the score calculation, and hence the possibility that cardiovascular risk by this indicator was underestimated cannot be ruled out. 

These results are significant for this population and, to our knowledge, this is the first study to evaluate cardiovascular risk using all these indicators.

## Conclusions

Xavante people have high cardiovascular risk according to indicators such as HDL-c, TG/HDL-c ratio, BMI, WC, HW and glucose levels.

Considering that CVD patients are initially asymptomatic, and that CVDs are important causes of morbidity and mortality, the present analysis of cardiovascular risk factors may be used as a basis for the planning of preventive measures and early treatment to minimize the impact of these diseases on this population.
